# Harmful Effects of Hyperoxia in Postcardiac Arrest, Sepsis, Traumatic Brain Injury, or Stroke: The Importance of Individualized Oxygen Therapy in Critically Ill Patients

**DOI:** 10.1155/2017/2834956

**Published:** 2017-01-26

**Authors:** Jean-Louis Vincent, Fabio Silvio Taccone, Xinrong He

**Affiliations:** ^1^Department of Intensive Care, Erasme Hospital, Université Libre de Bruxelles, 1070 Brussels, Belgium; ^2^Department of Intensive Care, Sun Yat-sen University Cancer Center, Guangzhou 510060, China

## Abstract

The beneficial effects of oxygen are widely known, but the potentially harmful effects of high oxygenation concentrations in blood and tissues have been less widely discussed. Providing supplementary oxygen can increase oxygen delivery in hypoxaemic patients, thus supporting cell function and metabolism and limiting organ dysfunction, but, in patients who are not hypoxaemic, supplemental oxygen will increase oxygen concentrations into nonphysiological hyperoxaemic ranges and may be associated with harmful effects. Here, we discuss the potentially harmful effects of hyperoxaemia in various groups of critically ill patients, including postcardiac arrest, traumatic brain injury or stroke, and sepsis. In all these groups, there is evidence that hyperoxia can be harmful and that oxygen prescription should be individualized according to repeated assessment of ongoing oxygen requirements.

## 1. Introduction

**Figure 1:**
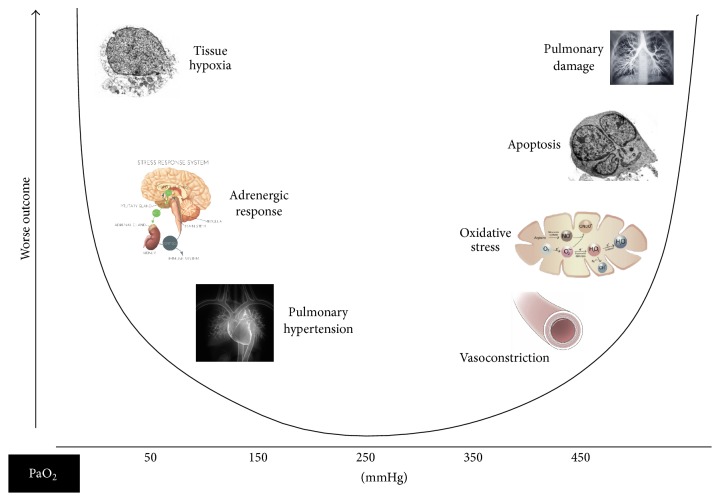
Schematic showing U-shaped association of PaO_2_ with outcome.

Oxygen is the third most abundant element in the universe and essential for life, but it was only officially “discovered” in the early 1770s separately by the British-born theologian, Joseph Priestly, and the Swedish apothecary, Carl Scheele [1, 2]. It took another few years for its role in respiration to be identified by the French chemist, Antoine-Laurent Lavoisier, who also gave it its name [1, 2]. Introduced into anaesthetic practice in the 1930s, oxygen is now one of the most widely used “drugs” in hospitalized patients. In a point-prevalence study conducted in 40 intensive care units (ICUs) in Australia and New Zealand in 2012, 59% of patients were receiving mechanical ventilation; among those not receiving mechanical ventilation, 86% were receiving oxygen via nasal cannulas, facial masks, or noninvasive ventilation [3]. However, although oxygen therapy clearly has important benefits in many patients, we have become increasingly aware of the potential harmful effects of high oxygenation concentrations in blood and tissues (Figure 1). In a retrospective study comparing mortality rates and PaO_2_ levels in mechanically ventilated ICU patients, de Jonge et al. reported a U-shaped relationship with increased mortality rates at low and high PaO_2_ [4]. The potential risks of hyperoxia, with a focus on recent clinical evidence in specific groups of critically ill patients (Table 1), will be the emphasis of this short narrative review.

## 2. Effects of Hyperoxia

Adequate cellular oxygenation is essential for normal cell function, and a low SaO_2_ is life-threatening, especially in acute conditions. Providing supplementary oxygen will increase oxygen delivery in hypoxaemic patients, thus supporting cell function and metabolism and limiting organ dysfunction. However, in patients who are not hypoxaemic, supplemental oxygen will increase oxygen concentrations into hyperoxaemic ranges. Although human beings may be exposed to hypoxia, for example, when at altitude or as a result of pulmonary disease, we are never exposed to hyperoxia, so that supplying extra oxygen to individuals who are not hypoxaemic is always a “nonphysiological” event.

Hyperoxia is associated with multiple effects in different organ systems. It can directly damage tissues via the production of reactive oxygen species (ROS) in excess of physiological antioxidant defence capabilities [5], leading to increased cell death by apoptosis and increased release of endogenous damage-associated molecular pattern molecules (DAMPs) that stimulate an inflammatory response, notably in the lungs [6] and vasoconstriction, likely as a result of reduced nitric oxide levels [7]. Orbegozo Cortés et al. recently reported that normobaric hyperoxia in healthy volunteers was associated with reduced capillary perfusion as assessed using sublingual side-stream dark field (SDF) video-microscopy [8]. It has been suggested that these vasoconstrictive effects may provide a means of protecting cells from the harmful effects of high PaO_2_ [9].

### 2.1. After Cardiac Arrest or Myocardial Infarction

Given the associated vasoconstriction and increased ROS release, hyperoxia may be particularly harmful after cardiac arrest [10]. Experimental and observational data have given conflicting results regarding the effects of hyperoxia in this setting [10]. In a retrospective analysis of data from 6326 postcardiac arrest patients admitted to ICUs in 120 US hospitals between 2001 and 2005, patients with hyperoxia (defined as PaO_2_ of ≥300 mmHg) on arrival in the ICU had higher mortality rates than those with normoxia or hypoxia; in multivariable analysis, hyperoxia exposure was an independent predictor of in-hospital death (odds ratio 1.8 [95% CI 1.5–2.2], *p* < 0.001) [11]. In an analysis of a registry database, severe hyperoxia (as identified by a PaO_2_ > 300 mmHg) was associated with increased mortality in postcardiac arrest patients, whereas moderate or “probable” hyperoxia (PaO_2_ 101–299 mmHg) was not [12]. In a retrospective cohort study, patients managed according to a conservative oxygen approach after cardiac arrest, targeting an pulse oximetry oxygen saturation (SpO_2_) of 88–92% had shorter lengths of ICU stay, although there were no differences in neurological outcomes [13]. In a multicentre trial conducted in 441 patients with ST-elevation myocardial infarction, patients with an SpO_2_ > 94% were randomized to receive 8 L/min of oxygen or no supplemental oxygen from arrival of paramedics until transfer to the cardiac care unit. Patients treated with oxygen had an increased rate of recurrent myocardial infarction, an increase in the frequency of cardiac arrhythmias, and an increase in myocardial infarct size at 6 months on magnetic resonance imaging [14]. These results do not support the use of routine supplemental oxygen after cardiac arrest or myocardial infarction. A randomized multicentre study is ongoing in Sweden aiming to randomize 6,600 patients with suspected acute myocardial infarction and SpO_2_ ≥ 90% to either 6 L/min of supplemental oxygen for 6 to 12 hours or room air [15].

### 2.2. In Traumatic Brain Injury and Stroke

Reduced cerebral oxygenation after brain injury is associated with impaired mitochondrial function and reduced metabolic rate and may be associated with an increased risk of secondary brain damage [16, 17]. Treating such patients with hyperoxia may, therefore, be expected to have beneficial effects on outcomes. However, clinical studies have given conflicting results. In a retrospective study of more than 3000 patients with traumatic brain injury (TBI), hypoxaemia, and extreme hyperoxaemia (PaO_2_ > 487 mmHg) on admission were both associated with worse outcomes; a PaO_2_ value of 110–487 mmHg was considered optimal in this study [18]. Similar findings were reported by a more recent retrospective study in 1547 patients with TBI, with both low and high admission PaO_2_ levels independently associated with worse outcomes [19]. In a long-term outcomes study after TBI, although there was a significant association between hyperoxaemia and a decreased risk of 6-month mortality in univariate analysis, in multivariable analysis, hyperoxaemia was not independently associated with outcome [20]. In a small randomized trial, 68 patients with severe TBI received either 80% or 50% oxygen via mechanical ventilation in the first 6 hours after the TBI. Patients in the hyperoxia group had better outcomes at 6 months as assessed using the Glasgow Outcome Scale than patients in the normoxia group [21]. A planned larger study to compare treatment with an FiO_2_ of 0.4 or 0.7 in patients with TBI was terminated because of slow recruitment (NCT01201291). Interestingly, in a prospective study of 30 patients monitored with a brain tissue oxygen sensor, Vilalta et al. reported that a hyperoxia challenge was associated with improved cerebral metabolism only in patients with reduced metabolism at baseline [22].

Evidence from studies in patients with stroke is also conflicting. Lang et al. reported no effect on 3-month neurological outcomes or mortality of moderate hyperoxaemia during the first 24 hours after ICU admission in patients after subarachnoid haemorrhage [31], and Young et al. similarly reported no association between worst PaO_2_ in the first 24 hours after ICU admission and mortality in patients with acute ischaemic stroke [36]. However, other observational studies have reported detrimental effects on short and longer term outcomes in different groups of stroke patients [28, 30]. A study randomizing patients to room air or supplemental oxygen administered at 30–45 L/min for 8 hours was terminated early because of more deaths in the oxygen group (NCT00414726). In a pilot study comparing oxygen supplementation for 72 h via nasal cannulae with room air in 289 patients with acute stroke, there was a small improvement in neurological recovery at one week [37], but there were no significant differences between the groups at 6 months [38], findings supported by the larger Stroke Oxygen Study in more than 8000 patients [39].

### 2.3. In Sepsis and Septic Shock

The use of hyperoxia in patients with sepsis is also controversial [40]. Sepsis is already associated with increased formation of ROS, believed to play a role in the tissue damage and organ dysfunction seen during sepsis. Hyperoxaemia is known to stimulate release of ROS and could therefore further worsen organ function in these patients. In a rat caecal ligation and puncture model, hyperoxia was associated with increased inflammatory cytokine release and organ dysfunction compared to normoxia [41]. However, in other animal models of sepsis, hyperoxia has been associated with improved haemodynamics and anti-inflammatory effects [42, 43]. And in a sheep model of sepsis, we showed that hyperoxia was associated with better haemodynamics and organ function compared to normoxia (unpublished data). In experimental human endotoxaemia, hyperoxia had no effect on levels of inflammatory mediators [44], and in a small observational study of patients with suspected sepsis in the emergency department, there were no significant differences in mortality rates between hyperoxic and normoxic patients [32]. A clinical trial in patients with sepsis randomized to hyperoxia or normoxia and hypertonic or isotonic saline in a 2 × 2 factorial design was stopped prematurely because of increased mortality rates in the hyperoxia and hypertonic saline arms (NCT01722422). Two randomized studies, one comparing supplemental oxygen titrated to different PaO_2_ targets (105–135 mmHg versus 60–90 mmHg, O_2_-ICU study, NCT02321072) and one comparing supplemental oxygen at 15 L/min to no supplemental oxygen (NCT02378545), are currently ongoing and should provide final answers as to whether or not patients with sepsis may benefit from hyperoxia.

### 2.4. In Mixed ICU Patients

Use of liberal oxygen therapy is frequent in critically ill patients [45] and severe hyperoxaemia (PaO_2_ > 200 mmHg) is associated with higher mortality rates [35]. Interestingly, in three ICUs in the Netherlands, more than 70% of ICU patients had PaO_2_ levels that were higher than the upper limits identified by the ICU clinicians treating them [46]. Several studies have now compared so-called conservative oxygen strategies targeting lower PaO_2_ or SpO_2_ values with conventional oxygen administration. Panwar et al. compared target SpO_2_ values of 88–92% and ≥96% in 103 ICU patients and reported no significant differences between the groups in terms of organ function or ICU and 90-day mortality [33]. In the Oxygen-ICU study, which was terminated early, 434 patients were randomized to receive supplemental oxygen to maintain PaO_2_ at 70–100 mmHg (SpO_2_ 94–98%) or to be managed conventionally allowing PaO_2_ to reach 150 mmHg (SpO_2_ 97–100%) [34]. Patients in the conservative group had lower ICU mortality than those in the conventional group (relative risk 0.57 [95% CI 0.37–0.9]; *p* = 0.01).

## 3. Conclusions

For many years, the known risks of hypoxia and less known adverse effects of hyperoxia have led to many patients receiving liberal oxygenation to avoid hypoxaemia at all costs. Although good quality data remain limited, results from the latest clinical studies seem to suggest that hyperoxaemia may be associated with worse outcomes in some critically ill patients (Table 1). The trend is therefore moving towards a more conservative approach to oxygenation aimed at maintaining SpO_2_ targets at 95–97%, although the optimal PaO_2_ level has not yet been defined and will likely change during the course of a patient's illness. Indeed, there may be a time window during which patients may benefit from higher oxygen levels [47]. Further well-designed randomized controlled trials in carefully selected groups of patients may help provide some definitive answers to these questions. As with other areas of intensive care management, oxygen therapy should be individualized. Patients who are hypoxaemic clearly need to receive supplemental oxygen, but ongoing requirements need to be reassessed on a regular basis to limit any risks associated with hyperoxia.

## Figures and Tables

**Table 1:** Some recent clinical studies on the risks of hyperoxia after cardiac arrest or myocardial infarction, in traumatic brain injury, stroke, sepsis, and mixed ICU patients.

References	Study design	Hyperoxia measurements	Main finding
After cardiac arrest or myocardial infarction			

Kilgannon et al. 2010 [11].	Retrospective cohort study, 120 hospitals, 6326 patients (nontraumatic cardiac arrest)	First PaO_2_ in the first 24 hours. Hyperoxia: PaO_2_ ≥ 300 mmHg	Hyperoxia was associated with an increased hospital mortality compared with either hypoxia or normoxia (OR 1.8 [1.5–2.2])

Bellomo et al. 2011 [23]	Retrospective cohort study, 125 ICUs, 12108 patients (nontraumatic cardiac arrest)	Worst PaO_2_ in first 24 h. Hyperoxia: PaO_2_ ≥ 300 mmHg Normoxia: PaO_2_ 60–300 mmHg	Hyperoxia group had a higher hospital mortality than normoxia (OR 1.2 [1.1–1.6])

Kilgannon et al. 2011 [24]	Retrospective cohort study, 120 hospitals, 4459 patients (nontraumatic cardiac arrest)	Highest PaO_2_ in the first 24 hours	A 100 mmHg increase in PaO_2_ was associated with a 24% increase in mortality risk (OR 1.24 [1.18 to 1.31])

Ranchord et al. 2012 [25]	Pilot randomized controlled trial, single-centre, 136 patients with STEMI	Patients randomized to receive high-concentration (6 L/min) or titrated oxygen (to achieve oxygen saturation 93%–96%) for 6 hours after presentation	No differences in number of deaths in the two groups (relative risk 0.5, 95% CI 0.05–5.4, *p* = 0.56)

Janz et al. 2012 [26]	Retrospective analysis of a prospective cohort study, single-centre 170 patients (cardiac arrest treated with mild therapeutic hypothermia)	Highest PaO_2_ in first 24 h.	Increased hospital mortality for every 100 mmHg increase in PaO_2_ (OR 1.49 [1.03, 2.14])

Lee et al. 2014 [27]	Retrospective cohort study, single-centre, 213 patients (cardiac arrest treated with therapeutic hypothermia)	Average PaO_2_ between ROSC and the end of rewarming. Hyperoxia: PaO_2_ > 157 mmHg Normoxia: PaO_2_ 117–135 mmHg	V-shaped association between PaO_2_ and poor neurologic outcome at hospital discharge (OR 6.47 [1.68, 24.91])

Stub et al. 2015 [14]	Prospective, randomized, controlled trial, 9 hospitals, 441 patients with STEMI	Patients with an SpO_2_ > 94% were randomized to receive 8 L/min of oxygen or no supplemental oxygen from arrival of paramedics until transfer to the cardiac care unit	An increased rate of recurrent myocardial infarction, an increase in the frequency of cardiac arrhythmias, and an increase in myocardial infarct size at 6 months on magnetic resonance imaging in the supplement group

Elmer et al. 2015 [12]	Retrospective analysis of a high-resolution database, single-centre, 184 patients postcardiac arrest	Mean hourly exposure in first 24 h. Normoxia: PaO_2_ 60–100 mmHg; Moderate hyperoxia: PaO_2_ 101–299 mmHg; Severe hyperoxia: PaO_2_≥ 300 mmHg	Severe hyperoxia was associated with decreased survival (OR 0.83 [0.69–0.99] per hour exposure); moderate hyperoxia was not associated with survival but with improved SOFA score 24 h (OR 0.92 [0.87–0.98])

Eastwood et al. 2016 [13]	Retrospective before-after nested cohort study, single-centre, 50 patients postcardiac arrest	Conservative oxygenation: SpO_2_ 88–92%	Conservative group had a shorter ICU length of stay; no difference in the proportion of survivors discharged from hospital with good neurological outcome compared to conventional group

In traumatic brain injury (TBI) and stroke			

Davis et al. 2009 [18]	Retrospective cohort study, 5 trauma centres, 3420 moderate-to-severe patients	Extreme hyperoxia: first PaO_2_ > 487 mmHg	A PaO_2_ value of 110–487 mmHg was considered optimal. Extreme hyperoxia had an independent association with decreased survival (OR 0.50 [0.36, 0.71]) compared to optimal range

Brenner et al. 2012 [19]	Retrospective study, single-centre, 1547 severe TBI patients	Mean PaO_2_ in first 24 h hospital admission: Hyperoxia: PaO_2_ > 200 mmHg Normoxia: PaO_2_ 100–200 mmHg	Both low and high PaO_2_ had increased mortality. Patients with hyperoxia had higher hospital mortality (OR 1.50 [1.15–1.97]) and lower discharge GCS scores at discharge (OR 1.52 [1.18–1.96])

Raj et al. 2013 [20]	Retrospective nested cohort analysis, 5 hospitals, 1116 ventilated moderate-to-severe TBI patients	Worst PaO_2_ in first 24 h ICU admission: Hyperoxia: PaO_2_ > 100 mmHg Normoxia: PaO_2_ 75–100 mmHg	Hyperoxia had no independent relationship with in-hospital mortality (OR 0.94 [0.65–1.36]) and 6-month mortality (OR 0.88 [0.63–1.22])

Rincon et al. 2014 [28]	Retrospective cohort, 84 ICUs, 2894 stroke patients	PaO_2_ in the first 24 hours. Hyperoxia: PaO_2_ ≥ 300 mmHg Normoxia: PaO_2_ 60–300 mmHg	Hyperoxia was independently associated with in-hospital mortality (OR 1.22 [1.04–1.48])

Rincon et al. 2014 [29]	Retrospective cohort study, 61 hospitals, 1212 ventilated TBI patients	Hyperoxia: PaO_2_ > 300 mmHg Normoxia: PaO_2_ 60–300 mmHg	Hyperoxia was associated with a higher in-hosptial case fatality (OR 1.5 [1.02–2.4])

Jeon et al. 2014 [30]	Prospective, observational cohort database analysis, single-centre, 252 patients (subarachnoid haemorrhage)	PaO_2_ AUC by observation time until delayed cerebral ischemia (DCI). Hyperoxia: PaO_2_ ≥ 173 mmHg (upper quartile)	Hyperoxia group had a higher incidence of DCI (OR 3.16 [1.69 to 5.92]) and poor outcome (modified Rankin Scale 4–6 at 3 months after subarachnoid haemorrhage) (OR 2.30 [1.03 to 5.12])

Quintard et al. 2015 [17]	Retrospective analysis of a database, single-centre, 36 severe TBI patients	Hyperoxia: PaO_2_ > 150 mmHg	Hyperoxia was associated with increased cerebral microdialysis glutamate, indicating cerebral excitotoxicity

Lang et al. 2016 [31]	Retrospective analysis using 2 databases, 432 ventilated patients (subarachnoid haemorrhage)	Time-weighted average PaO_2_ during the first 24 hours Low PaO_2_ < 97.5 mmHg; Intermediate PaO_2_ 97.5–150 mmHg; High PaO_2_ >150 mmHg	Patients with an unfavorable outcome had significantly higher PaO_2_, but high PaO_2_ has no effect on 3-month neurological outcomes (OR 1.09 [0.61–1.97]) or mortality (OR 0.73 [0.38–1.40])

In sepsis			

Stolmeijer et al. 2014 [32]	Prospective pilot study, 83 sepsis patients in emergency department, single-centre	PaO_2_ after 5 min of a VentiMask 40% with 10 L O_2_/min. Hyperoxia: PaO_2_ > 100 mmHg	Of the hyperoxic patients, 8% died in hospital versus 6% with normoxia

In mixed ICU patients			

de Jonge et al. 2008 [4]	Retrospective observational study, 50 ICUs, 36307 ventilated patients	Worst PaO_2_ in first 24 h. Hyperoxia: PaO_2_ ≥ 123 mmHg (upper quintile) compared with PaO_2_ between 67 and 80 mmHg	In-hospital mortality was linearly related to FiO_2_ value and had a U-shaped relationship with PaO_2_. Hyperoxia had a higher mortality (OR 1.23 [1.13–1.34])

Panwar et al. 2016 [33]	Pilot randomized controlled trial, 4 ICUs, 103 patients	Conservative oxygenation: SpO_2_ 88–92% Liberal oxygenation: SpO_2_ ≥ 96%	No significant differences in measures of new organ dysfunction, or ICU or 90-day mortality (OR 0.77 [0.40–1.50])

Girardis et al. 2016 [34]	Open-label randomized trial, single-centre, 434 patients	Conservative oxygenation: PaO_2_ 70–100 mmHg (SpO_2_ 94–98%) Conventional oxygenation: PaO_2_ > 150 mmHg (SpO_2_ 97–100%)	Patients in the conservative group had lower ICU mortality (RR 0.57 [0.37–0.9]) and fewer episodes of shock, liver failure, and bacteraemia

Helmerhorst et al. 2017 [35]	Observational cohort study, 3 ICUs, 14441 ventilated patients	First PaO_2_ at ICU admission Mild hyperoxia: PaO_2_ 120–200 mmHg Severe hyperoxia: PaO_2_ > 200 mmHg	Severe hyperoxia was associated with higher mortality rates and fewer ventilator-free days in comparison to both mild hyperoxia and normoxia Time spent in hyperoxia had a linear and positive relationship with hospital mortality

OR: odds ratio; SOFA: sequential organ failure assessment; ROSC: return of spontaneous circulation; DIC: delayed cerebral ischemia; AUC: area under the curve; STEMI: ST-segment elevated myocardial infarction.
